# Parathyroid hormone ameliorates temporomandibular joint osteoarthritic‐like changes related to age

**DOI:** 10.1111/cpr.12755

**Published:** 2020-03-10

**Authors:** Chen Cui, Liwei Zheng, Yi Fan, Jun Zhang, Ruoshi Xu, Jing Xie, Xuedong Zhou

**Affiliations:** ^1^ State Key Laboratory of Oral Diseases National Clinical Research Center for Oral Diseases West China Hospital of Stomatology Sichuan University Sichuan China; ^2^ School of Stomatology Kunming Medical University Kunming China

**Keywords:** cellular senescence, cyclin‐dependent kinase inhibitor P16^INK4A^, marrow mesenchymal stem cells, osteoarthritis, temporomandibular joint disorders

## Abstract

**Objectives:**

Ageing could be a contributing factor to the progression of temporomandibular joint osteoarthritis (TMJ OA), whereas its pathogenesis and potential therapeutic strategy have not been comprehensively investigated.

**Materials and methods:**

We generated ageing mouse models (45‐week and 60‐week; 12‐week mice as control) and intermittently injected 45‐week mice with parathyroid hormone (PTH(1‐34)) or vehicle for 4 weeks. Cartilage and subchondral bone of TMJ were analysed by microCT, histological and immunostaining. Western blot, qRT‐PCR, ChIP, ELISA and immunohistochemical analysis were utilized to examination the mechanism of PTH(1‐34)’s function.

**Results:**

We showed apparent OA‐like phenotypes in ageing mice. PTH treatment could ameliorate the degenerative changes and improve bone microarchitecture in the subchondral bone by activating bone remodelling. Moreover, PTH inhibited phosphorylation level of Smad3, which can combine with p16^ink4a^ gene promoter region, resulting in reduced senescent cells accumulation and increased cellular proliferation of marrow mesenchymal stem cells (MSCs). ELISA also showed relieved levels of specific senescent‐associated secretory phenotype (SASP) in ageing mice after PTH treatment.

**Conclusions:**

In summary, PTH may reduce the accumulation of senescent cells in subchondral bone by inhibiting p16^ink4a^ and improve bone marrow microenvironment to active bone remodelling process, indicating PTH administration could be a potential preventative and therapeutic treatment for age‐related TMJ OA.

## 1 INTRODUCTION

Temporomandibular joint osteoarthritis (TMJ OA), a progressive cartilage degradation and subchondral bone alterations, is accompanied with pain, joint clicking, and limitation or deviation in the mandibular range of motion.^1^, ^2^ It has been shown that 14.56% of mainland Chinese patients with TMJ disorders had radiographic signs of OA, which has brought enormous challenges to social economics.^3^ The most common risk factors of TMJ OA include gender, mechanical stress and age.^4^, ^5^ Ageing has been considered as a very important reason. The TMJ, like the knee and other joints, develops OA with ageing.^6^ The signs of degenerative changes on the TMJ articular surfaces increases with advancing age (50% in aged group vs 28% in young group).^7^, ^8^ In the mandibular condylar cartilage, ageing causes reduction in chondrocytes number and viability, decrease in matrix synthesis, and the appearance of OA lesions along the articular surface of the joint.^9^ In the subchondral bone region, ageing animals exhibited significantly reduced bone volume.^10^

Temporomandibular joint osteoarthritis causes changes in cartilage, subchondral bone, synovial membrane and other tissues.^11^, ^12^ Recently, an increasing evidence supports the significance of subchondral bone in the pathogenesis of TMJ degenerative disease.^13^, ^14^ Subchondral bone locates below the layer of calcified cartilage to provide mechanical support. Its alterations antedate the changes in articular cartilage during OA progression.^15^, ^16^ Subchondral bone remodelling is a progression throughout life in TMJ because of occlusion movement. To maintain a physical remodelling process, the activity of osteoclasts, which cause bone resorption, and osteoblasts, which are responsible for bone formation, must be precisely coordinated. During bone remodelling, factors released locally to mediate bone marrow microenvironment, which influences the recruitment and fate of bone marrow mesenchymal stem cells (MSCs) for new bone formation.^17^, ^18^, ^19^, ^20^, ^21^ In the early progress of OA, the remodelling of subchondral bone is upregulated, whereas the activity of osteoblasts cannot catch with osteoclasts, resulting in decreased bone mineral density.^22^, ^23^, ^24^ Some reasons contribute to this impaired balance, for instance, decreased number of MSCs, and reduced capacity of proliferation or differentiation which occurs with ageing.^25^, ^26^

Parathyroid hormone (PTH), an 84‐amino‐acid polypeptide, is a systemic hormone that regulates calcium homoeostasis by a direct action on bone and kidney. It has been revealed that intermittent injection of PTH (1‐34) not only increases apparent bone mass, but also improves microarchitecture of bone such as trabecular number and connectivity by modulating bone marrow microenvironment and increasing the number of osteoblasts in ageing mice.^27^, ^28^ Furthermore, studies demonstrated a phenomenon that PTH had an effect on cell cycle progression.^29^, ^30^ While intermittent treatment of PTH has been used to prevent the progression of OA in knee or spine osteoarthritis both in animal and humans,^31^, ^32^ not much is known about the specific function of PTH administration in TMJ OA. Therefore, in the current study, we set out to address the effect and underlying mechanism of intermittently injection of PTH (1‐34) in ageing mice with TMJ OA‐like changes. We have found that PTH (1‐34) treatment ameliorated the degenerative changes in TMJ condyles and improved subchondral bone microarchitecture by increasing the number of osteoblasts in ageing mice. Moreover, PTH inhibited the expression of p16^ink4a^, a senescence biomarker as well as relieved the expression of specific senescent‐associated secretory phenotype (SASP), indicating PTH administration could ameliorate TMJ OA‐like alterations related to age.

## 2 MATERIALS AND METHODS

### 2.1 Animal

Seven‐week‐old C57BL/6J male mice were purchased from Chengdu Dossy Biological Technology Co., Ltd and housed in animal centre of West China Hospital, and were randomly selected at the age of 12, 45 or 60 weeks (n = 5 per group) to sacrifice. For the PTH treatment experiment, 45‐week‐old mice were assigned into two groups (n = 10 per group), subcutaneous injected with human PTH (1‐34) (40 μg/kg per day, Bachem, Inc) or an equivalent volume of vehicle (1 mmol/L acetic acid in phosphate buffered saline (PBS)), 5 days per week, for 4 weeks.^33^ All animal experiments were carried out in accordance with the approved guidelines of Ethical Committees of the West China School of Stomatology, Sichuan University and the State Key Laboratory of Oral Diseases.

### 2.2 MicroCT analysis

Temporomandibular joint samples were harvested at indicated times and scanned using a microCT scanner (μCT50; SCANO, Switzerland) at a voltage of 50 kVp, a current of 200 µA and a resolution of 3.0 µm per pixel. The images were reconstructed and performed three‐dimensional histomorphometry based on a previous report.^10^ Briefly, two cubic regions of interest (each 100 × 100 × 100 µm^3^) were defined from the middle of the centre and posterior third of the condylar subchondral cancellous bone. The selected regions were analysed to determine the trabecular bone volume fraction (BV/TV), trabecular thickness (Tb. Th), trabecular number (Tb. N) and trabecular separation (Tb. Sp).

### 2.3 Histologic and immunologic staining

Samples were fixed in 4% paraformaldehyde for 3 days. After decalcification in 20% EDTA (pH 7.5), samples were processed, embedded in paraffin and cut into 4‐μm sections using a microtome (Leica, RM2235, Germany). Haematoxylin/eosin (HE) and Safranin‐O staining were performed to assess condyle histomorphology. The Mankin and Osteoarthritis Research Society International (OARSI) scores were used to assess histologic grading of cartilage degeneration.^34^ Tartrate‐resistant acid phosphatase (TRAP) staining, using a standard protocol (Sigma‐Aldrich), was used to detect osteoclasts.

Immunologic analyses were performed using an Anti‐Rabbit/Mouse ABC Staining Kit (Vector Laboratories). The staining procedures were followed as manufacturer's instruction. The following antibodies were used: MMP13 (1:100, Santa Cruz Biotechnology), COL X (1:200, Abcam), COL I (1:200, Abcam), OSX (1:200, Abcam), RUNX2 (1:200, Abcam), CTSK (1:100, Santa Cruz Biotechnology), pSMAD3 (1:200, Abcam), pCREB (1:100, Assay biotechnology), CDKN2A/p16^INK4a^ (1:200, Abcam), Ki67 (1:200, Cell Signaling Technology) and SOX9 (1:200, Abcam). TUNEL staining was performed using the in situ cell death detection kit (Roche Diagnostics, Mannheim, Germany), and DAPI (Vector) was used as counterstaining. After mounting, the slides were photographed with an Olympus BX53 microscope (Olympus, Japan).

For quantitative analysis, condylar subchondral cancellous bone was regarded as regions of interest, and cell counting in bone marrow was conducted by a blinded observer with ImageJ software. OSX^+^, TRAP^+^ and TUNEL^+^ cells were normalized by the bone surface, and p16^+^, pSMAD3^+^ and pCREB^+^ were normalized by the number of total cells. Three different sections were used from each sample, and three different samples were used for each group.

### 2.4 Serologic test

Blood was obtained by cheek pouch puncture after fasting for 4 hours. Inflammatory factors were measured by using Milliplex Multiplex Assays (EMD Millipore). Inflammatory factors we examined include GM‐CSF, IL‐1α, IL‐2, IL‐4, IL‐5, IL‐6, IL‐10, IL‐12, IL‐13, IL17A, KC, LIX, MCP‐1, MCP‐2 and TNF‐α. All measurements were performed in duplicate.

### 2.5 Isolation of MSCs from mouse mandibular and cell culture

Mesenchymal stem cells from mouse mandible were isolated as described by previously.^35^ Briefly, we collected mandible from 12‐ or 45‐week‐old mice and removed the attached soft tissues and teeth. Digest with 3 mg/mL collagenase type I (Worthington Biochem) and 4 mg/mL dispase II (Roche Diagnostic) for 60 minutes at 37°C. Cells cultured in α‐MEM (Gibco) supplemented with 10% foetal calf serum (Gibco) and 1% penicillin–streptomycin (HyClone) at 37°C, 5% (v/v) CO_2_.

Cells >80% confluence were digested with trypsin (HyClone), and P3 cells were inducted by osteogenic induction medium (10 mmol/L β‐glycerophosphate and 50 μg/mL ascorbic acid), treated with human PTH (1‐34) (100 nmol) or PBS.^33^ Alkaline phosphatase (ALP) (Beyotime, China) staining and alizarin red staining (ARS) (Cyagen, China) were performed 7 days or 14 days after osteogenic induction, respectively. Moreover, cells were induced with adipocyte differentiation medium (500 μm IBMX, 1 μm Dexamethasone, 5 μg/mL Insulin and 1 μm Rosiglitazone) for two days and base medium (5 μg/mL Insulin and 1 μm Rosiglitazone) for 4 days. Oil red O staining was performed at the 6th day. To form three‐dimensional cartilage balls, MSCs were plated at 1.5‐mL microcentrifuge tube with 3 × 10^6^ cells/tube and cultured in chondrogenic differentiation media (Cyagen) for 14 days. Then, cartilage balls were collected for staining.

OMSCs from 45‐week mice treated with PTH or vehicle for 7 days in vitro were performed with SA‐β gal staining (Cell Signaling Technology). The percentage distribution of cell cycle phases (G0/ G1, S and G2/M) of viable cells was further determined by using the Cell Cycle Assay Kit (Beyotime).

### 2.6 Chromatin immunoprecipitation assay (ChIP)

Cells were fixed with 1% PFA, and ChIP assay was performed according to manufacturer's guidelines (Cell Signaling Technology). DNA that co‐precipitated with Smad3 (Cell Signaling Technology) was analysed using primers specific for the 5′ gene regulatory regions of p16 (F = GTCACACGACTGGGCGATT, R = GTTGCCCATCATCATCACCT and F = GATGACTTCACCCCGTCACT, R = AACACCCCTGAAAACACTGC/GT CCCTCCTTCCTTCCTCTG). After 40‐55 cycles of amplification, fragments produced from each primer set were examined and confirmed for their predicted molecular masses on EtBr‐stained 2% agarose gels. Fifty‐five cycles of amplification were used for negative control PCRs.

### 2.7 Quantitative RT‐PCR and Western blot

Total RNA was extracted using TRIzol reagent (Invitrogen) according to instruction. The purity of RNA was tested by measuring the absorbance (NanoDrop ND‐1000, Thermo Fisher Scientific) and reverse transcribed into cDNA using the PrimeScript RT Reagent Kit (Takara). The expressions of genes were measured with Bio‐Rad iQ5 real‐time PCR detection system, with β‐actin for normalization. The primers for target genes were listed in Table 1.

**Table 1:** Primer of target genes

	Forward primer	Reverse primer
ALP	CACGGCCATCCTATATGGTAA	GGGCCTGGTAGTTGTTGTGA
OSX	CTCCTTGGTGGGACATGC	GTAGGCAGCTGGGGGTTC
OCN	CTGACCTCACAGATCCCA AGC	TGGTCTGATAGCTCGTCACAAG
RUNX2	TCCACAAGGACAGAGTCAGATTACAG	CAGAAGTCAGAGGTGGCAGTGTCATC
COL1	GCGCTAAAGGTGCCAATG	AGCACCAGGTTCACCACTG
DMP1	TGTCCTGTGCTCTCCCAGT	TTCTTCTGATGACTCACTGTTCG
FGF23	TACTTGTCGCAGAAGCATCAC	GGCGAACAGTGTAGAAATGCAG
P16	GCTCAACTACGGTGCAGATTC	GCACGATGTCTTGATGTCCC
P21	CCTGGTGATGTCCGACCTG	CCATGAGCGCATCGCAATC
IL‐6	TAGTCCTTCCTACCCCAATTTCC	TTGGTCCTTAGCCACTCCTTC
TNF‐α	CCCTCACACTCAGATCATCTTCT	GCTACGACGTGGGCTACAG
β‐Actin	AAGGCCAACCGTGAAAAGAT	GTGGTACGACCAGAGGCATAC

Protein was extracted by whole cell lysis assay (KeyGEN), and the concentrations were evaluated by enhanced BCA protein assay kit (Beyotime). Equal amount of protein samples was separated by sodium dodecyl sulphate–polyacrylamide gel electrophoresis (Bio‐Rad Laboratories) and transferred onto a polyvinylidene fluoride membrane (Millipore). Following incubation in specific antibodies, we probed proteins by enhanced chemiluminescence kit (Bio‐Rad Laboratories). Bands were quantified by ImageJ software. Specific antibodies included CREB (Assay biotechnology, 1:1000), pCREB (Assay biotechnology, 1:1000), CDKN2A/p16^INK4a^ (Abcam, 1:1000), pSMAD3 (Abcam, 1:1000) and Tubulin α (Signalway Antibody, 1:2000).

### 2.8 Statistics

All experiments were performed independently in triplicate, and data (expressed as mean ± SEM) from a representative experiment are shown. For statistical analysis, SPSS 22.0 analysis software (IBM) was used. Two‐group comparisons were evaluated by unpaired two‐tailed Student's *t* tests, and multiple comparisons were performed by one‐way analysis of variance (ANOVA) with Bonferroni post hoc test. The level of significance was set at *P* values < .05.

## 3 RESULTS

### 3.1 TMJ OA‐like phenotype related to age

**Figure 1:**
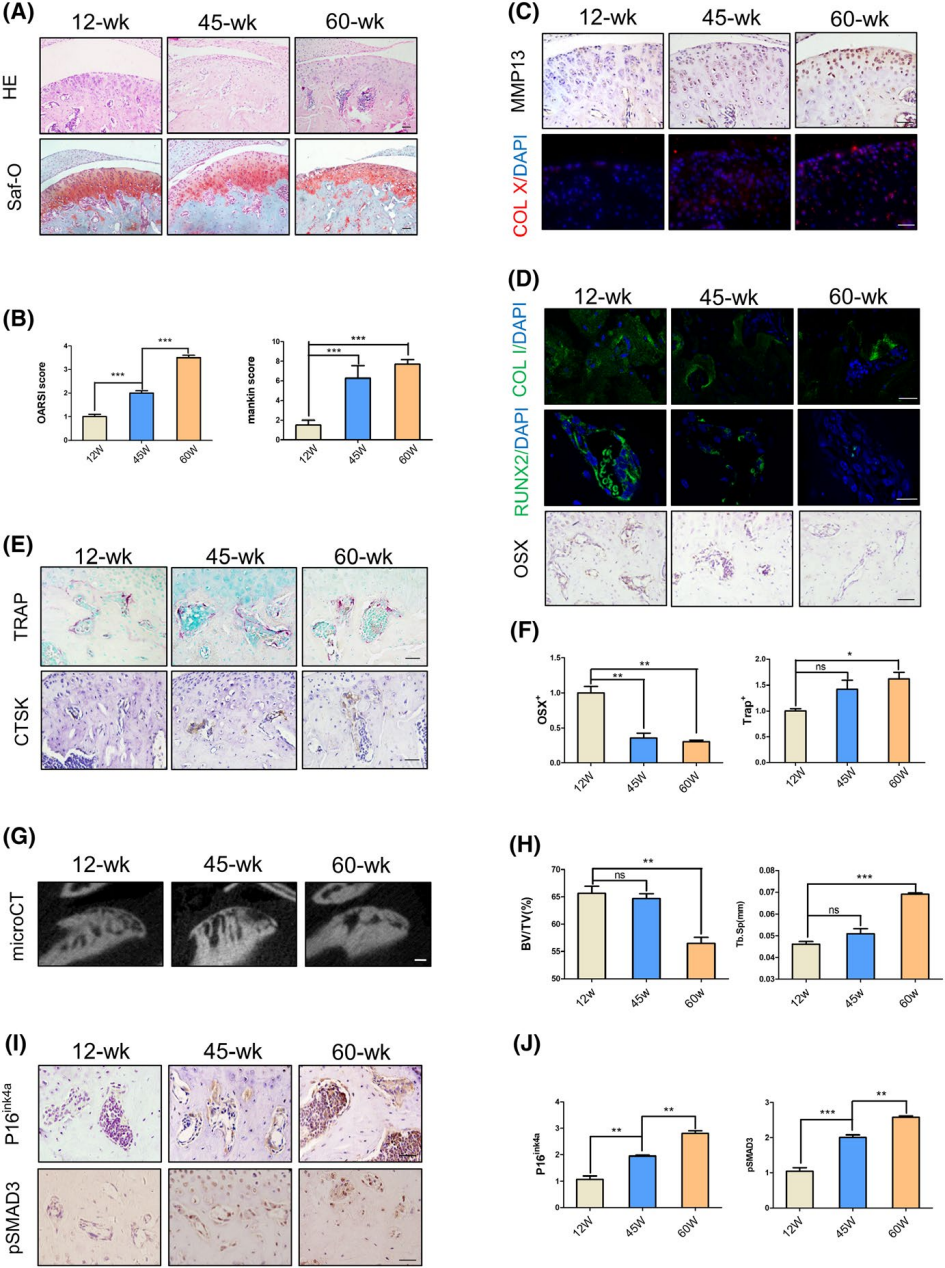
A degeneration phenotype was exhibited in temporomandibular condylar with ageing. A, HE and Safranin‐O staining images showing condylar cartilage layer of 12 wks, 45 wks or 60 wks. B, OA score (including OARSI and Mankin score) at different time points of mice condyle. C, Immunohistochemical and immunofluorescence analyses of metallomatrix protease 13 (MMP13) and collagen X (Col X) in the cartilage layer. D,E, Immunohistochemical and immunofluorescence (Osterix, Runx2, collagen I and cathepsin K) and tartrate‐resistant acid phosphatase (TRAP) staining in subchondral bone. F, Quantitative analysis of Osx^+^ and TRAP^+^ cell numbers. G,H, Representative μCT images and quantitative analysis. BV/TV (%), bone volume fraction; Tb.Sp (mm), trabecular separation. I,J, Immunohistochemical images showing expression of P16ink4a and pSMAD3 positive cells and quantitative analysis in subchondral bone. Scale bars, 50 μm. N = 5 per group. *P < .05, ***P < .005. Bars depict the mean ± SD and analysed by one‐way ANOVA followed by Tukey's test

We first examined the changes in mandibular condyle phenotype during ageing by histological staining. HE staining indicated that, when compared to 12‐week‐old mice, condyle surface irregularities and the number of hypertrophic chondrocytes significantly increased in 45‐ and 60‐week‐old mice. It was notable that surface clefts were observed in 60‐weeks mice. In addition, proteoglycan loss was visualized by Safranin‐O staining. A significant increase in OA scores (OARSI and Mankin score) was found in 45, 60‐weeks mice, indicating a degenerative phenotype in TMJ with ageing (Figure 1A,B). Furthermore, immunologic analysis showed that the expression of matrix metalloproteinase 13 (MMP13) and collagenase X (Col X) was significantly higher in 45‐ and 60‐weeks relative to 12‐weeks mice in cartilage layer (Figure 1C).

In subchondral bone region, immunofluorescent staining of collagenase I (Col I) and Runx2 showed downregulated expression levels in ageing mice compared with 12‐weeks controls. The number of Osx^+^ osteoprogenitors was statistically decreased in 45‐ and 60‐weeks mice, with the minimum number in 60‐weeks group (Figure 1D,F). TRAP staining and cathepsin K (CtsK) expression suggested that osteoclasts number was significantly increased with ageing (Figure 1E,F). MicroCT analysis further revealed that 60‐weeks mice had significantly decreased bone volume (%, BV/TV) and increased trabecular space (mm, Tb.Sp) (Figure 1G,H).

P16^ink4a^ is the cyclin‐dependent kinase (CDK) inhibitor and a senescence biomarker.^36^, ^37^ Recent studies revealed that p16^ink4a^‐positive cells accumulated in numerous tissues, including subchondral bone with ageing.^38^ Similarly, we observed significantly increased expression levels of p16^ink4a^ in 45‐ and 60‐weeks mice. Moreover, we have previously suggested abnormal activation of TGF‐β signalling in the condylar cartilage and subchondral bone in ageing mice.^10^ In accordance with this phenotype, the phosphorylation level of Smad3 was significantly increased in 45‐ and 60‐weeks mice when compared to 12‐weeks controls (Figure 1I,J).

### 3.2 PTH directly induces phosphorylation of CREB (pCREB) in mandibular condyle

**Figure 2:**
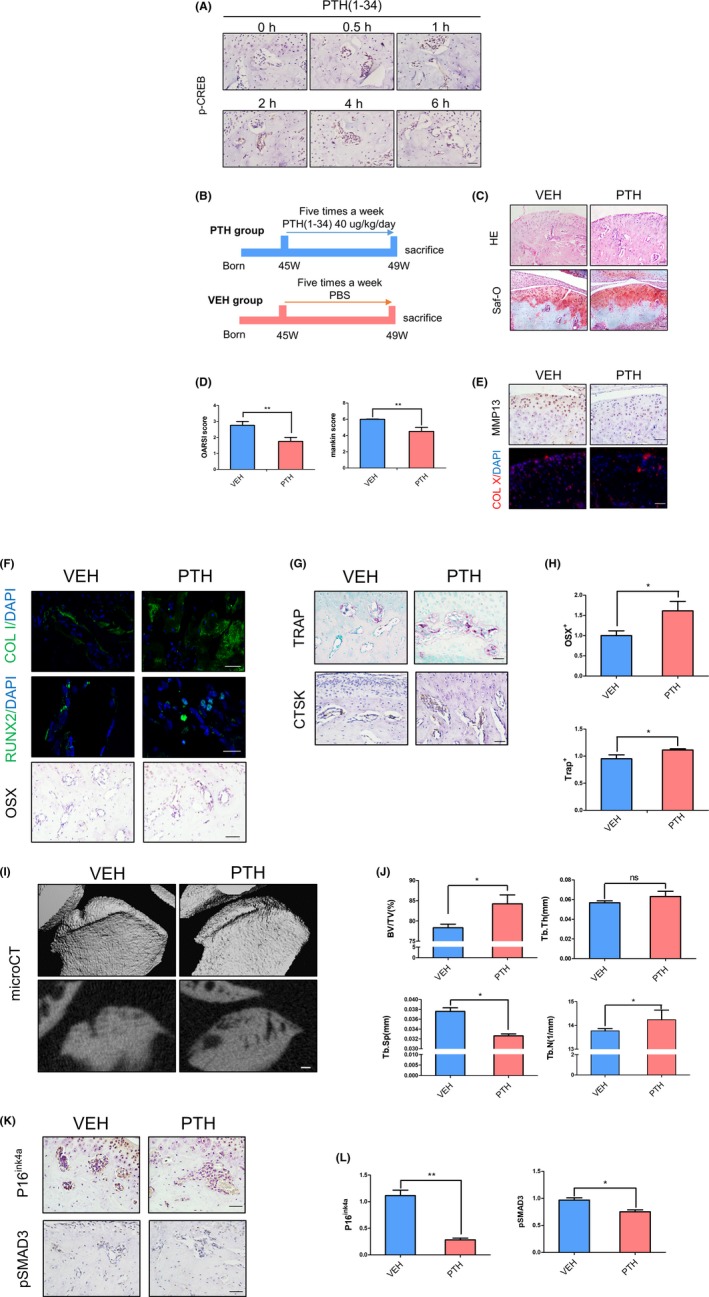
Intermittent administration of PTH (iPTH) attenuates condyle degeneration in ageing mice. A, Immunostaining of subchondral bone showing pCREB‐positive cells in bone marrow at different time points with PTH treatment. B, Schematic plot of PTH treatment strategy. C,D, HE and Safranin‐O staining images and OA score in 45‐wk‐old mice with iPTH or vehicle. E, Immunohistochemical and immunofluorescence analyses of MMP13 and Col X in the cartilage layer. F‐H, Immunohistochemical and immunofluorescence (Osterix, Runx2, collagen I and cathepsin K) and TRAP staining in subchondral bone, and quantitative analysis of Osx^+^ and TRAP^+^ cell numbers. I,J, μCT images and quantitative analysis in two groups. BV/TV (%), bone volume fraction; Tb.Th (mm), trabecular thickness; Tb.Sp (mm), trabecular separation; Tb.N (1/mm), trabecular number. K,L, Immunohistochemical (P16^ink4a^ and pSMAD3) staining images and cell counting in different groups. Scale bars, 50 μm. N = 5 per group. *P < .05. Bars depict the mean ± SD and analysed by unpaired two‐tailed Student's *t* tests

We next asked whether subcutaneous injection of PTH (1‐34) activates its downstream signalling in mandibular condyle subchondral bone. It has been found that PTH can stimulate downstream intracellular signalling, including cAMP/PKA signalling by virtue of phosphorylation of CREB.^39^ Therefore, we performed immunostaining of phosphorylated CREB (pCREB) and visualized that pCREB expression was significantly induced in subchondral bone region at 30 minutes and peaked at 1 hour after PTH (1‐34) single injection. Its expression was returned to normal after 6 hours post‐injection. These data revealed the direct signalling of PTH in subchondral bone region (Figure 2A).

### 3.3 Intermittent administration of PTH attenuates condyle degeneration

We performed 4‐week intermittent PTH (1‐34) injection to 45‐week‐old mice (Figure 2B). Histology analysis and scores revealed that PTH (1‐34) administration significantly ameliorated surface irregularities and proteoglycan loss in condyle surface. Clefts and heterotopic osteogenesis in cartilage layer were also rescued in PTH‐injected group (Figure 2C,D). MMP13 and Col X expression levels were also downregulated after PTH (1‐34) treatment, indicating that OA phenotype in cartilage was ameliorated by PTH treatment (Figure 2E).

In subchondral bone, expression levels of Col I, Runx2 and Osx were upregulated in PTH‐injected group (Figure 2F,H). And TRAP and CstK staining revealed an upregulation in osteoclast number in PTH‐injected group (Figure 2G,H). Moreover, microCT indicated that subchondral bone was smoother in PTH‐injected group, accompanied by increased bone volume (%, BV/TV) and trabecular number (1/mm, Tb.N) as well as decreased trabecular space (mm, Tb.Sp) (Figure 2I,J). Intriguingly, p16^ink4a^ and pSmad3‐positive cell accumulation in bone marrow were significantly reduced after PTH (1‐34) treatment (Figure 2K,L).

### 3.4 Mouse orofacial bone marrow‐derived MSCs (OMSCs) have self‐renew and multi‐lineage differentiation potential

**Figure 3:**
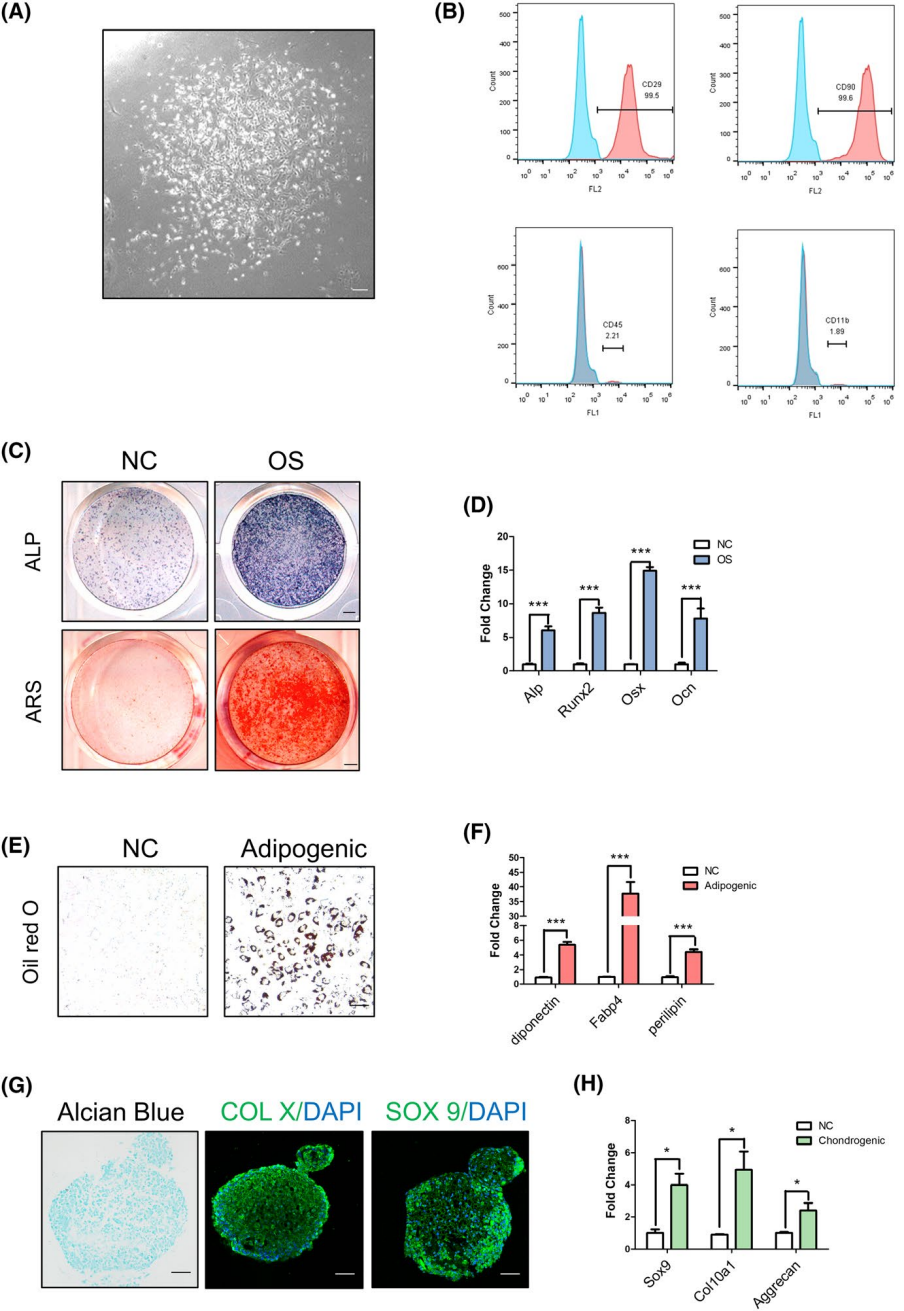
Mesenchymal stem cells isolated from mandible have multi‐lineage differentiation potential. A, Light microscope image of primary OMSCs. B, Flow cytometry showing OMSCs surface molecule. C,D, Alkaline phosphatase (ALP) staining, alizarin red staining (ARS) and quantitative analysis of osteogenic gene expression after osteogenic induction. E,F, Oil red O staining detecting the lipid accumulation, and gene expression analysis after adipogenic induction. G,H, Alcian blue and immunofluorescence (Col X and Sox 9) staining images in cartilage balls and gene expression after chondrogenic induction. Scale bars, 50 μm. N = 3 per group. *P < .05, ***P < .005. Bars depict the mean ± SD and analysed by unpaired two‐tailed Student's *t* tests

We have previously demonstrated PTH functions to direct long bone MSCs cell fate.^40^ Recent studies discovered OMSCs resided in mandibular bone region, which exhibited several distinct traits when compared to long bone MSCs.^41^ To further examine the function of PTH on mandibular bone in vitro, we isolated mouse mandibular OMSCs. Observed with the light microscope, OMSCs showed a typical fibroblast‐like morphology (Figure 3A). Flow cytometric analysis was performed to examine the surface maker expressions—positive for MSCs markers and negative for hematopoietic markers (Figure 3B). Then, OMSCs were cultured in osteogenic, adipogenic or chondrogenic media. Histology and gene expression analysis proved the potential of OMSCs to differentiate into osteoblasts, adipocytes and chondrogenic cells in vitro (Figure 3C‐H).

### 3.5 PTH (1‐34) induces osteogenic differentiation in young and ageing OMSCs

**Figure 4:**
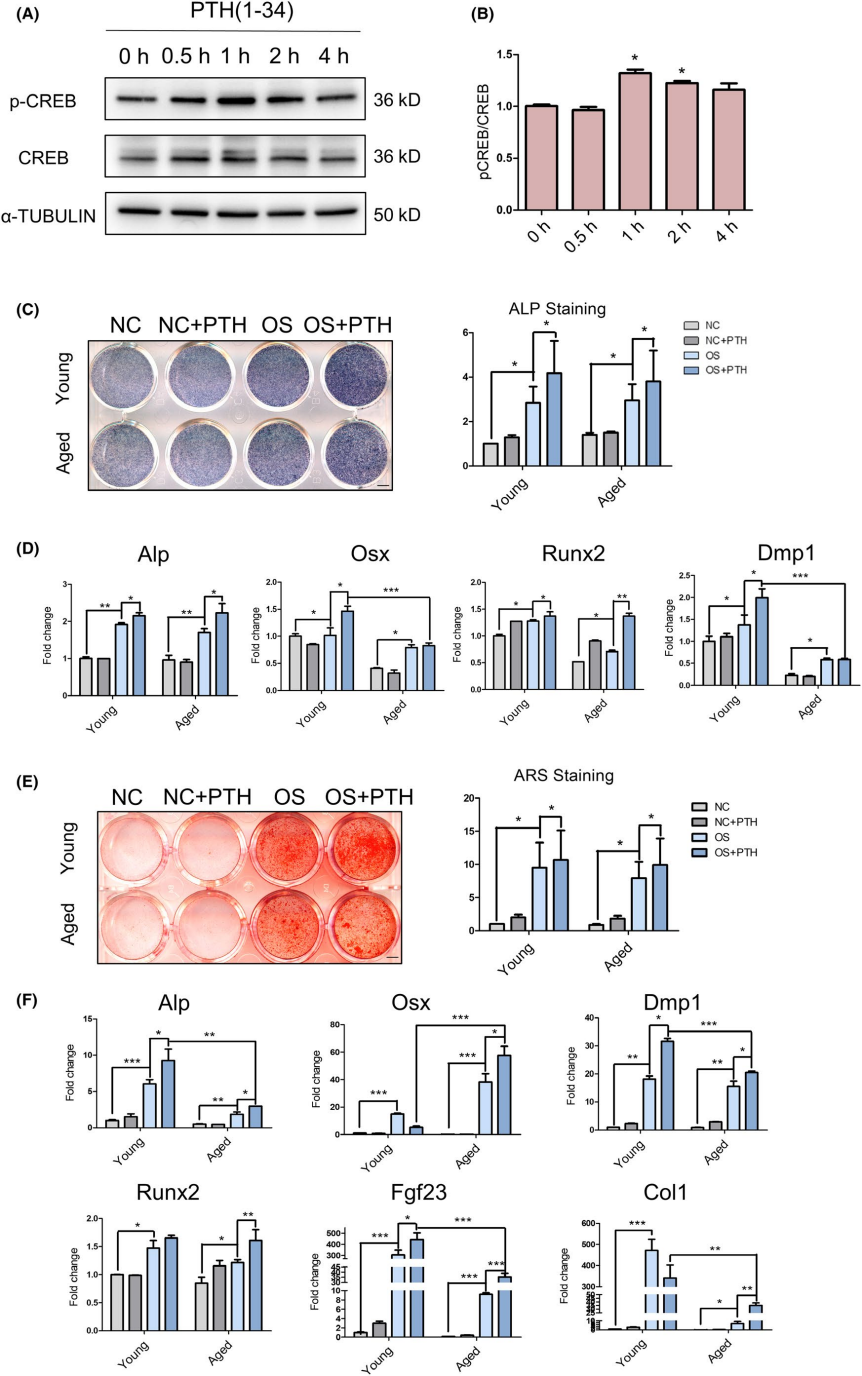
OMSCs derived from ageing mice have an intense potential of osteogenesis with PTH treatment. A,B, Western blot analysis showing pCREB and CREB levels in OMSC after treated with PTH at different time points, and quantitative analysis. C,D, ALP staining and osteogenic‐related gene expression analysis after osteogenic induction for 7 days with PTH or vehicle. E,F, ARS and osteogenic‐related gene expression analysis after osteogenic induction for 14 d with PTH or vehicle. N = 3 per group. *P < .05, ***P < .005. All data were expressed as the mean ± SD

OMSCs of young and ageing mice were utilized to determine the effect of PTH (1‐34) treatment in vitro. We performed single administration of PTH (1‐34) and showed increased pCREB expression in OMSCs at 1 hour post‐induction (Figure 4A,B), confirming activated cAMP/PKA signalling upon PTH treatment. We next induced OMSCs under osteogenic medium for 7‐14 days. ALP staining and ARS as well as gene expression analysis revealed that osteogenesis was successfully induced in young and ageing OMSCs when compared to their undifferentiated control (NC) counterparts as expected. Osteogenic medium (OS)‐induced group treated with PTH (1‐34) was associated with more intense ALP staining and ARS when compared to OS‐alone group. Interestingly however, aged‐OMSCs demonstrated less mineralized nodules when compared to young‐OMSCs group at the same condition (Figure 4C,E). Gene expression analysis also suggested similar results, showing relatively reduced osteoblast and osteocyte markers (Alp, Osx, Runx2, Dmp1, Col1 and FGF23) in aged‐OMSCs (Figure 4D,F).

### 3.6 PTH regulates p16^ink4a^ expression in OMSCs through TGF‐β signalling

**Figure 5:**
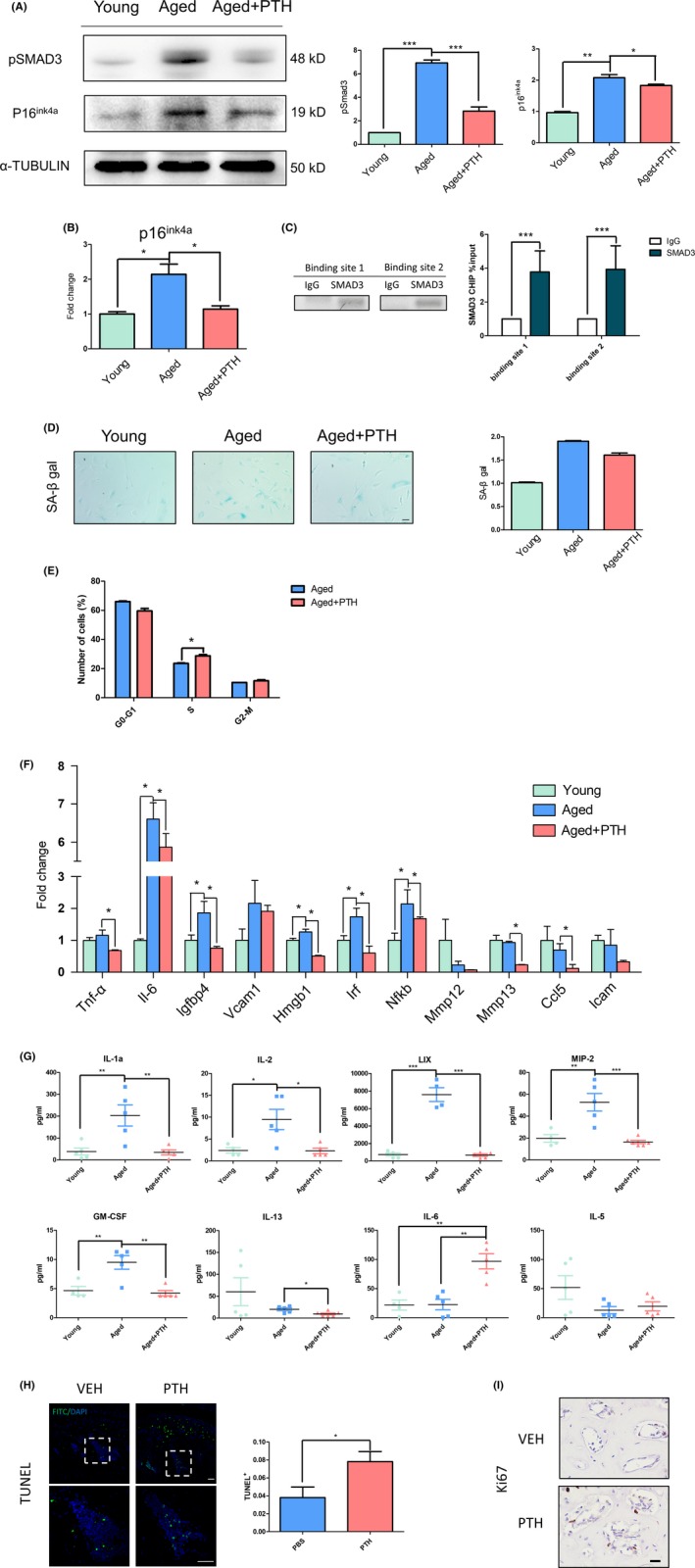
PTH inhibited p16^ink4a^ expression by decreasing pSMAD3 level in aged‐OMSCs. A, Protein levels of pSMAD3 and p16^ink4a^ in young cells, aged cells and aged cells with PTH for 14 days. B, Gene expression analysis of p16^ink4a^ in three groups. C, CHIP‐PCR and CHIP‐qPCR results of two sites in p16 ^ink4a^ promoter were shown. D, Senescence‐associated β‐galactosidase (SA‐β‐gal) staining and positive cells counting. E, Percentage distribution of cell cycle phases. F, Gene expression of senescence‐associated secretory phenotype (SASP) in OMSCs. G, Inflammatory factors in mice serum were measured. H, TUNEL staining was performed in vehicle and PTH treatment group to detect apoptotic cells. I, Immunohistochemical analyses of ki67 in the bone marrow. N = 3 per group. *P < .05. All data were expressed as the mean ± SD

We have previously shown that pSMAD3 expression was activated along with ageing while PTH (1‐34) treatment reduced its activity in vivo (Figures 1I and 2K). Indeed, Western blot analysis demonstrated that pSmad3 protein expression was dramatically increased in aged‐OMSCs, while PTH (1‐34) treatment significantly inhibited pSmad3 expression (Figure 5A). Furthermore, qRT‐PCR and Western blot analysis indicated that aged‐OMSCs had higher p16^ink4a^ expression than young cells and PTH (1‐34) treatment significantly downregulated its expression at both transcript and protein levels (Figure 5A,B).

In addition, it has been proved that Smad3 can assemble in the promoter region of p16^ink4a^ to regulate its expression in muscle satellite cells,^42^ and p16^ink4a^ regulates cell cycle progression by preventing S phase entry.^36^, ^37^ Thus, we sought to analyse the relationship between Smad3 and p16^ink4a^ in OMSCs using ChIP assay. Using primers specific for 5′ promoter regulatory regions of p16^ink4a^ by qRT‐PCR and PCR, the results showed Smad3 can combine with the promoter region of p16^ink4a^ in OMSCs (Figure 5C). The percentage distribution of cell cycle phases (G0/G1, S and G2/M) of aged‐OMSCs revealed that more cells enter into S phase after PTH (1‐34) treatment (23.5 ± 0.35% vs 28.5 ± 0.92%) (Figure 5E).

### 3.7 PTH regulates senescence of OMSCs and subchondral bone marrow microenvironment

It has previously suggested that p16^ink4a^ is one of the biomarkers in cellular senescence. Senescent cells express SASP leading to alterations in bone marrow microenvironment.^43^ Therefore, we analysed the number of senescent cells and the expression of SASP factors. SA‐β gal staining appeared increased tendency in aged‐OMSCs; however, the number of senescent cells reduced after 7‐day PTH (1‐34) administration (Figure 5D). Transcript levels of 11 established SASP factors showed that 5 out of the 11 SASP genes were upregulated in aged‐OMSCs, while 8 out of the 11 SASP genes downregulated by PTH (1‐34) treatment (Figure 5F). In vivo, we inspected the levels of inflammatory factors in serum of aged mice after 4‐week PTH (1‐34) injection or vehicle, compared with young mice. The results showed that inflammatory factor (IL‐1a, IL‐2, LIX, MIP‐2, GM‐CSF) levels increased in aged mice serum, while suppressed after 4‐week PTH (1‐34) injection (Figure 5G). Moreover, TUNEL assay suggested that intermittent PTH (1‐34) injection statistically increased apoptotic cells (Figure 5H). Proliferative cells were then examined by the Ki67 expression levels, showing upregulated number of proliferative cells in PTH‐injected mice (Figure 5I).

## 4 DISCUSSION

The present study provides evidence that intermittent PTH (1‐34) administration could ameliorate the degenerative alterations associated with TMJ OA in ageing mice. It could improve subchondral bone microarchitecture by accelerating bone remodelling and modulate senescence phenotype through inhibiting p16^ink4a^ and SASP expression. While age has always been regarded as a very important aetiologic agent for OA,^5^ the pathogenesis of TMJ OA development is more complex than we thought because of age‐related changes in the musculoskeletal system.^7^, ^44^, ^45^ We first generated 45‐ and 60‐weeks old mice as ageing models. In line with this, we observed that OA scores (the OARSI and Mankin score) and the number of Col X‐ and MMP13‐positive cells increased with ageing, which were phenotypes of cartilage degeneration.^46^, ^47^ The cartilage layer in 60‐weeks old mice even formed surface clefts and bone matrix like‐tissue. Moreover, numerous studies have shown that uncoupled bone remodelling by osteoclasts and osteoblasts in subchondral bone priors to cartilage degeneration, resulting in breakdown of the overlying cartilage, and ultimately leading to TMJ OA lesions.^48^, ^49^ Therefore, we have next focused on the subchondral bone region, with a particular interest in osteoclast‐ and osteoblast‐mediated bone coupling. The results displayed increased number of osteoclasts while decreased number of osteoblasts in condylar along with ageing. This feature of inevitable bone loss with ageing has also been suggested by other studies showing different position of bone, frequently causing osteoporosis and osteoarthrosis.^50^ These results suggested that the condyles of 45‐ and 60‐weeks‐old mice were in the progress of TMJ OA, and 45‐weeks‐old mice were chosen for subsequent analysis as they presented as an early‐onset TMJ OA model.

Parathyroid hormone is a major endocrine regulator of mineral ion homoeostasis in extracellular calcium and phosphate levels.^51^ Continuous PTH exposure results in catabolic effects, while intermittent exposure is associated with anabolic effects.^52^ Thus, intermittent PTH (1‐34) (teriparatide) treatment has been used for the treatment of osteoporosis.^53^, ^54^ Moreover, researchers also found that intermittent treatment of PTH can prevent the progression of knee or spine osteoarthritis,^31^, ^32^ but not much is known about the specific function of PTH administration in TMJ OA. In our study, we performed 4‐weeks intermittent hypodermic injection of PTH (1‐34) in 45‐weeks animals, and then, we found both osteogenic and osteolysis capabilities were upregulated in subchondral bone region in 45‐weeks mice. Intriguingly, subchondral bone volume of PTH‐treated group was significantly higher than that of vehicle group, supporting the tenet that osteogenic ability was more pronounced than osteolysis capability. What's more, an increased trabecular number and a decreased trabecular space reminded us of a comparative normal structure of subchondral bone after 4‐week administration. Of note, OA scores (the OARSI and Mankin score) and abnormal expression of Col X and MMP13 were partially corrected in cartilage layer after PTH (1‐34) administration, possibly due to amelioration of condylar subchondral bone degeneration.

Studies demonstrated that PTH (1‐34) can apparently increase long bone MSC proliferation and osteoblast differentiation, whereas its effects on OMSCs are still unknown.^55^ OMSCs located in mandibular bone marrow, which developed from migrating cranial neural crest cells, distinct from long bone MSCs in terms of differentiation traits.^41^, ^56^ Aged OMSCs displayed less potential of osteogenesis in vitro*;* however, a significant activation of osteogenic differentiation is shown after administrated with PTH (1‐34), indicating accelerated osteogenic differentiation of OMSCs contributed to observed increased subchondral bone volume to some extent. Furthermore, PTH has an inhibition effect on pSmad3 by reducing the number of TGF‐β receptors in membrane.^17^, ^57^ And in muscle satellite cells, Smad3 can aggregate at the promoter region of p16^ink4a^ to accelerate its expression.^42^ Thus, we performed the ChIP assay using OMSCs and confirmed that Smad3 has a similar effect on p16^ink4a^ promoter, suggesting PTH treatment may function to suppress pSmad3, leading to the subsequent reduction in p16^ink4a^.

Some studies found that senescent cells depend on pro‐survival pathways to defend themselves against their pro‐apoptotic microenvironment,^58^ and the accumulation of aged cells not only losses proliferation potential, but also creates their own microenvironment, a low‐grade inflammation, to affect adjacent cells by SASP.^59^ Thus, clearing senescent cells would be a new strategy to reduce senescent cell burden.^60^, ^61^ Recent studies focused on senolytic drugs gave us new avenue to defend age‐related diseases, such as frailty, cardiac dysfunction, etc.^62^ The main effect of senolytic drugs was to improve both health and lifespan near the end of life by clearing senescent cells in all tissues and organs.^61^ Treatment of senolytic drugs may be feasible to alleviate age‐related TMJ OA by selectively inducing apoptosis of senescent cells. PTH, on the other hand, can targets various types of cells, including bone marrow stromal cells, osteoprogenitors, T lymphocytes, osteocytes and osteoclasts, thus modulating bone marrow microenvironment.^17^, ^51^ In the present study, we found intermittent PTH (1‐34) treatment had an effect on bone marrow stromal cells to activate bone remodelling process. Moreover, the results showed relieved SASP expressions and inflammatory cytokine expression levels after PTH treatment. Therefore, PTH (1‐34) treatment may improve both bone marrow microenvironment and reduce the accumulation of senescent cells in therapeutic settings.

It has been found that senescent cells can defend themselves against apoptosis,^58^ as well as affect adjacent cells by senescent‐associated secretory phenotype.^59^ Therefore, it would be viable to promote senescent cells undergoing apoptosis process to reduce their burden.^60^, ^61^, ^63^ Our study showed increased apoptotic cells after PTH(1‐34) treatment, adding to a decreased number of senescent cells, indicating PTH may play a role in reducing senescent cells burden. Therefore, we hypothesized that PTH influences physiological functions of stem cells by reducing the surrounding senescent cells to maintain the microenvironment in subchondral bone marrow.

Temporomandibular joint osteoarthritis is one of the common temporomandibular disorders that limits the life quality of patients, and ageing plays a critical role in spontaneous TMJ OA. From our current investigation, we found that the condyle of 45‐ and 60‐weeks mice shows early changes in cartilage layer and subchondral bone related to TMJ OA phenotype. Intermittent PTH treatment can ameliorate the abnormal changes by stimulating bone remodelling in subchondral bone region, possibly by inducing osteogenic differentiation of resident OMSCs and enhancing osteoclasts activity. More importantly, we showed that PTH downregulated p16^ink4a^ expression and therefore reduced senescent cells accumulation and improved the senescent‐associated microenvironment, and as a result, ameliorated the occurrence of age‐related TMJ OA.

## CONFLICT OF INTEREST

The authors declare no competing financial interest.

## AUTHOR CONTRIBUTIONS

CC, XZ, LZ and JZ designed the research; CC, YF, JZ, RX and JX performed the experiments; CC, JZ and RX performed the in vivo experiments; CC, YF and JX performed the in vitro experiments; CC, YF, LZ and XZ analysed the data and wrote the manuscript; XZ and LZ supervised the project and revised the manuscript.

## Data Availability

No publicly available data or shared data are cited. Raw data were submitted in the system. Derived data supporting the findings of this study are available from the corresponding author (XZ) on request.
